# Sediment Disturbance Negatively Impacts Methanogen Abundance but Has Variable Effects on Total Methane Emissions

**DOI:** 10.3389/fmicb.2022.796018

**Published:** 2022-02-21

**Authors:** Annette Rowe, Megan Urbanic, Leah Trutschel, John Shukle, Gregory Druschel, Michael Booth

**Affiliations:** ^1^Department of Biological Sciences, University of Cincinnati, Cincinnati, OH, United States; ^2^Department of Earth Sciences, Indiana University-Purdue University Indianapolis, Indianapolis, IND, United States

**Keywords:** methanogenesis, bioturbation, greenhouse gas, freshwater sediment, methane ebullition

## Abstract

Methane emissions from aquatic ecosystems are increasingly recognized as substantial, yet variable, contributions to global greenhouse gas emissions. This is in part due to the challenge of modeling biologic parameters that affect methane emissions from a wide range of sediments. For example, the impacts of fish bioturbation on methane emissions in the literature have been shown to result in a gradient of reduced to enhanced emissions from sediments. However, it is likely that variation in experimental fish density, and consequently the frequency of bioturbation by fish, impacts this outcome. To explore how the frequency of disturbance impacts the levels of methane emissions in our previous work we quantified greenhouse gas emissions in sediment microcosms treated with various frequencies of mechanical disturbance, analogous to different levels of activity in benthic feeding fish. Greenhouse gas emissions were largely driven by methane ebullition and were highest for the intermediate disturbance frequency (disturbance every 7 days). The lowest emissions were for the highest frequency treatment (3 days). This work investigated the corresponding impacts of disturbance treatments on the microbial communities associated with producing methane. In terms of total microbial community structure, no statistical difference was observed in the total community structure of any disturbance treatment (0, 3, 7, and 14 days) or sediment depth (1 and 3 cm) measured. Looking specifically at methanogenic Archaea however, a shift toward greater relative abundance of a putatively oxygen-tolerant methanogenic phylotype (*ca.* Methanothrix paradoxum) was observed for the highest frequency treatments and at depths impacted by disturbance (1 cm). Notably, quantitative analysis of *ca.* Methanothrix paradoxum demonstrated no change in abundance, suggesting disturbance negatively and preferentially impacted other methanogen populations, likely through oxygen exposure. This was further supported by a linear decrease in quantitative abundance of methanogens (assessed by qPCR of the *mcrA* gene), with increased disturbance frequency in bioturbated sediments (1 cm) as opposed to those below the zone of bioturbation (3 cm). However, total methane emissions were not simply a function of methanogen populations and were likely impacted by the residence time of methane in the lower frequency disturbance treatments. Low frequency mechanical disruption results in lower methane ebullition compared to higher frequency treatments, which in turn resulted in reduced overall methane release, likely through enhanced methanotrophic activities, though this could not be identified in this work. Overall, this work contributes to understanding how animal behavior may impact variation in greenhouse gas emissions and provides insight into how frequency of disturbance may impact emissions.

## Introduction

Methane emissions from aquatic ecosystems including, lakes, reservoirs, and wetlands, have been shown to be a substantial (Cole et al., 2007; Bastviken et al., 2011; Deemer et al., 2016), and poorly constrained, contribution to global greenhouse gas production (Walter et al., 2007; Engram et al., 2020). Part of the challenge of constraining methane emissions is their inherently heterogeneous nature, both within and across locations (Musenze et al., 2014; Podgrajsek et al., 2014; Beaulieu et al., 2016). For example, within a single reservoir system, hotspots of methane production occur that are double or triple the rate of methane emissions of other regions in the reservoir (Berberich et al., 2020). Across locations, a range of physical and chemical parameters contribute to variation in methane production and consumption (Sobek et al., 2012; Beaulieu et al., 2014; Deemer et al., 2016; Grinham et al., 2018), but these general features often do not explain variation within a given system. Biological parameters, such as bioturbation, may offer insight into the variability of methane emissions both across sites and within the different habitats of a given system.

Bioturbation is the disruption of sedimentary deposits by living organisms (Kristensen et al., 2012). This can include small to large-scale disturbances and can range from the impacts of burrowing invertebrates to predators (e.g., fish) that feed on these invertebrates. Though the scale of effects vary, bioturbation often results in the mixing of geochemical or redox gradients and the introduction of oxygen to anoxic sediments (Bertics and Ziebis, 2009; Kristensen et al., 2012). In freshwater sediments, large-scale disruption of sediment by organisms such as benthic feeding fish can also enhance methane ebullition (Frei et al., 2007; Carey et al., 2018; Brown and Hershey, 2019), the release of gaseous methane bubbles that are formed and trapped in super saturating sediment conditions (Joyce and Jewell, 2003). Ebullition can result in increased total methane emissions from sediments, likely due to the escape of methane that would otherwise diffuse and be consumed by methane-oxidizing organisms (Frei and Becker, 2005; Frei et al., 2007; Datta et al., 2009). However, under some conditions, fish bioturbation has been shown to reduce methane emission, likely due to the exposure of anoxic sediments to oxygen which can inhibit methanogenic organisms (Oliveira Junior et al., 2019; Booth et al., 2021). Thus, it is not well understood how the degree or frequency of bioturbation impacts methane emissions through changing the balance of methanogenesis and methanotrophy in sediments and the physical perturbance of sediment to release methane bubbles *via* ebullition, nor how the structure of the microbial communities is impacted by bioturbation.

In most ecosystems, methane production (i.e., methanogenesis) is an anaerobic process performed by specific classes of Archaea that fall into seven orders: *Methanobacteriales, Methanococcales, Methanomicrobiales, Methanosarcinales, Methanocellales, Methanopyrales*, and *Methanomassiliicoccales*. Under standard conditions, methanogenesis conserves a small amount of energy and is traditionally thought to occur only in environments where more energetically favorable terminal electron acceptors, such as oxygen, nitrate, iron, and sulfate are absent (Zinder, 1993). In these anoxic environments, methanogenesis is a major driver of organic matter degradation and plays a critical role in decomposition. In addition to energetic constraints (and the likelihood of being out-competed for resources), the biochemistry involved in generating methane is highly sensitive to oxygen (Thauer et al., 2008). Many of the iron-sulfur proteins and cofactors involved in methanogenesis are damaged in the presence of oxygen, or can result in oxygen radical formation, which further constrains the growth of methanogens (Zinder, 1993). Oxygen tolerance in methanogens grown in culture or from sediment mesocosms has been shown to vary, ranging from exposure that results in complete loss of methanogenesis with prolonged recoveries post exposure (Yuan et al., 2009, 2011), to methanogens that contain and express genes to deal with oxygen exposure (i.e., superoxide dismutase) (Tholen et al., 2007). Even in tolerant organisms, oxygen presents a stress that can inhibit or reduce methane production.

**FIGURE 1:**
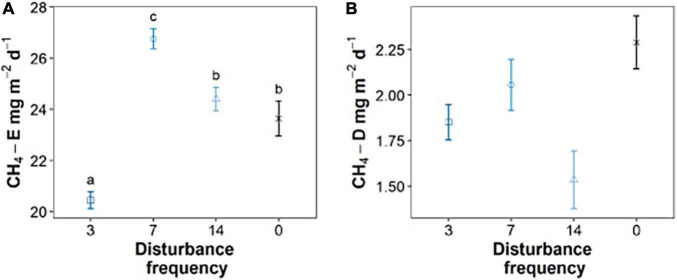
The impacts of intermediate sediment disturbance on rates of methane ebullition **(A)** and diffusion **(B)** for triplicate sediment mesocosms. Gas samples taken for three replicate mesocosms analyzed for each data point (*n* = 3). Means that do not share letters significantly differ (α = 0.05). Data from Booth et al. (2021).

This work investigates how sediment geochemistry and microbiology are impacted by mechanical disturbance (mimicking the sediment disruption caused by fish bioturbation of the sediment), building off our prior work investigating impacts on greenhouse gas emissions (Booth et al., 2021). Using sediments derived from Acton Lake (near Oxford, OH, United States), we constructed sediment microcosms that were exposed to different frequencies of disturbance to mimic different levels of bioturbation. Gas ebullition and diffusion measurements were taken for each treatment (Booth et al., 2021). Booth et al. (2021) observed enhanced methane ebullition at intermediate (7-day) disturbance frequencies (Figure 1A). No statistical difference was observed in terms of diffusion of methane related to disturbance frequency (Figure 1B). By measuring the gas volumes in our gas traps pre- and post-disturbance, we could link 65–75% of the gas ebullition in each disturbance treatment to disturbance events (Booth et al., 2021). Notably, other greenhouse gases were monitored in this work—specifically, CO_2_, N_2_O, and NO—but methane was the only greenhouse gas that demonstrated a statistically significant difference under varying bioturbation frequency (Booth et al., 2021). We hypothesized that oxygen exposure introduced by disturbance inhibited methanogenic microbial communities at the highest disturbance frequencies, resulting in lower total methane emissions. At intermediate frequencies, disruption of the sediment drove the escape of methane gas that would otherwise remain trapped in sediment and resulting in elevated methane emissions to lower frequency experiments. Here we combine microbial community analysis and quantitative PCR (qPCR) targeting the methanogenic community to investigate the corresponding impacts of sediment disturbance frequency on methanogenesis in disturbed sediments.

## Materials and Methods

### Sediment Microcosms and Disturbance Frequency

Sediment mesocosms [performed as part of Booth et al. (2021)] were constructed in 37.9 L glass aquarium tanks, using sediment from the littoral zone of Acton Lake—a shallow eutrophic reservoir in southwest Ohio, United States. Prior to addition to tanks, sediment was sieved through 6.35 mm mesh to remove large debris and then homogenized. Each tank received ∼ 19 L of sediment, filling the lower 15 cm of the tank. An additional 15 cm of the tanks were then filled with municipal tap water. The water column was aerated and gently mixed continuously using an aquarium pump and air stones in each tank. These conditions were meant to mimic the shallow edge of a reservoir where wind and currents help maintain an oxic water column. After construction, mesocosms were incubated for 60 days prior to any experimental treatment to allow sediment gradients to develop.

Temperature was monitored at 1-hour intervals using HOBO pendant temperature loggers (Onset Computer, Bourne, MA, United States). Other water quality parameters, including turbidity and dissolved oxygen, were monitored weekly over the course of these experiments using a YSI ProDSS (Yellow Springs, OH, United States) handheld multiparameter meter including an optical dissolved oxygen probe. Sediment disturbances that were meant to mimic the physical disruption of sediment caused by fish bioturbation were performed *via* a mechanical bioturbator that was constructed in house [see Supplementary Figure 1 or Booth et al. (2021)]. The bioturbator generates a circular 20 mm deep depression in sediment and uses a propeller to manually displace sediment upward in the affected region. Triplicate mesocosms were exposed to a different frequency of disturbance using the bioturbator over a 90-day period. Detailed methods are described in Booth et al. (2021). In brief, disturbances were performed using the bioturbator at 0-day (no disturbance), 3-day, 7-day, or 14-day intervals (*n* = 12). At each interval, a one-time disturbance treatment covering approximately 50% of the sediment surface area was executed. The patches for disturbance were selected randomly for each date using a random number generator and a numbered grid system.

### Oxygen Profiling

Oxygen profiles were measured using Au-amalgam microelectrodes (<1 mm diameter glass cylindrical housing) constructed as described previously (Smith et al., 2011; Houghton et al., 2019), controlled by a potentiostat (DLK-70, Analytical Instrument Systems). The electrode captured a redox profile of the various analytes across the sediment water interface by driving the microelectrode, mounted on an automated microprofiler (LabMan, Analytical Instrument Systems inc.) down at 2 mm increments, and performing cyclic voltammetry. Cyclic voltammetry is run at the Au-amalgam electrode surface relative to an Ag/AgCL reference electrode and Pt counter, at a scan rate of 1,000 mV/s between −0.1 and −1.8 V (vs. Ag/AgCL) with a 2 s deposition step at −0.1 V. This scan analyzes O_2_, Fe^2+^, chelated Fe^3+^, Mn^2+^, and H_2_S (Taillefert et al., 2000; Luther et al., 2001, 2008). Microelectrode data analysis and profile plotting were performed utilizing in-house developed R-based software that is publicly available on github.^1^

### Sediment Sampling and DNA Extraction

During construction of sediment microcosms, 5 g samples from sieved and homogenized sediments were taken from each tank prior to the start of the experiment and stored at −80^°^C for DNA analysis. After 90 days of treatment and methane quantification, sediment cores were taken in triplicate using a Wildco^®^ Hand Core Sediment sampler with core liners (2″ diameter 20″ length) for each tank and stored at −80°C prior to processing. To sample DNA, cores were extruded from their plastic lining using a core extruder. Approximately 1 g of sediment, taken from both 1 and 3 cm below the sediment-water interface, was collected aseptically from sediment cores for nucleic acid analysis. DNA was extracted from each 90 day sample and 4 time 0 samples using a Qiagen DNeasy PowerSoil Pro extraction kit (Qiagen, MD, United States) following manufacturers protocol with the following modifications: samples were bead beaten for 5 min, and final DNA was eluted in 50 μl of DNase/RNase free water. DNA concentrations were analyzed using a Qubit 4 Fluorometer and the dsDNA HS kit (Thermo Fisher Scientific, Waltham, MA, United States). All sample concentrations fell within 5–9 ng/μl.

### Quantitative PCR

Quantitative PCR was performed on a CFX96 Touch Real-Time PCR Detection system (Bio-Rad, Hercules, CA, United States). Calculation of *mcrA, pmoA*, and *ca*. Methanothrix paradoxum 16S rRNA gene copied numbers were performed on DNA extracts from all 1 and 3 cm samples and four samples from the pre-treated sediment. Degenerate primers for *mcrA—*which encodes a methanogen specific gene essential for methanogenesis—were mcrA-F (5′-GGTGGTGTMGGATTCACACARTAYGCWACAGC-3′) and mcrA-R (5′-TTCATTGCRTAGTTWGGRTAGTT-3′) (Luton et al., 2002). Primers for the particulate monooxygenase *pmoA* and *mmoX* were tested with general PCR on sediment samples (Mcdonald et al., 2008). Only the primers A189 F and A 682 R generated an amplicon in these sediments (data not shown). Quantification of *pmoA* gene copies was performed using the A189 (5′-GGNGACTGGGACTTCTGG-3′) and A682 (5′-GAASGCNGAGAAGAASGC-3′) primers (Holmes et al., 1995). Primers for the *ca*. Methanothrix paradoxum strain were designed in this work using the IDT DNA Sci tools, Real time qPCR Assay^2^ : Forward primer (5′-AGAGGTGAGAGGTACTTCAGG-3′) and reverse primer (5′-GGGTATCTAATCCGGTTCGTG-3′). For each nucleic acid sample, a qPCR reaction was run in triplicate 30-μl reactions including: 15 μl 2X Sybr Green Universal PCR Master Mix (Invitrogen), 3 μl of 10 μM of forward and reverse primers, and 3 μl of DNA template. Amplification conditions consisted of 40 cycles of 15 s at 95°C and 60 s at 55°C, followed by melt-curve analysis. Pure culture DNA from *Methanosarcina acetivorans* was used to generate an *mcrA* standard curve based on the weight of the genome and *mcrA* copy numbers per genome. Purified PCR products were used to generate a standard curve for both *pmoA* and *ca*. Methanothrix paradoxum analyses. Conversion of fluorescence data to gene copy numbers was performed using the Ct method run using the CFX Maestro Software (Bio-Rad) for analysis.

### Amplicon Sequencing and Microbial Community Analysis

Amplicon sequencing was performed by Novogene Co., Ltd. (Sacramento, CA, United States) using standard protocols for 16S rRNA sequencing. In brief, PCR was performed on DNA extracts using Phusion^®^ High-fidelity PCR master mix (New England Biolabs, United States) and universal 16S rRNA primers 515F and 806R targeting the V4 region (Caporaso et al., 2011). Samples were gel purified using the Qiagen Gel Extraction kit (Qiagen, Germany) and converted to libraries using the NEBNext^®^ Ultra™ DNA Library Prep Kit (New England Biolabs, Ipswich, MA, United States) using manufacturers recommendation for index codes. Library quality was assessed using a Qubit 2.0 Fluorimeter (Thermo Scientific, Waltham, MA, United States) and Agilent Bioanalyzer 2100 System pre-mixing, and subsequent sequencing using the Illumina platform with paired end 250 (PE250) chemistry using standard protocols.

Paired-end sequences were assembled using Flash (V1.2.7).^3^ Qiime 1.7 was used to assess quality and detect/remove chimeras using the UCHIME algorithm (Edgar et al., 2011). Organizational taxonomic unit (OTU) clustering was performed based on ≥ 97% sequence similarity in Qiime using the Uparse software (v7.0.1001). Unique OTUs were assigned taxonomy using the RDP classifier function and the GreenGene Database (Wang et al., 2007). OTU abundance was normalized using the sequence number corresponding to the sample with the least number of sequences. Raw OTU abundances are provided in the Supplementary Table 1. Sequence data is available through the NCBI sequence read archive under BioProject PRJNA766591.

Beta diversity was assessed by calculating the weighted and unweighted unifrac distances using the Qiime 1.7 software package. Hierarchical clustering was performed using the Unweighted Pair-group Method with Arithmetic Mean (UPGMA) in Qiime. Principal component analysis (PCA) and principal coordinate analysis (PCoA) based on the weighted and unweighted unifrac distance matrices were performed using R (version 2.15.3) and the FactoMineR and WGCNA packages along with ggplot2 (Langfelder and Horvath, 2008; Lê et al., 2008; Wickham, 2016).

### Data Analysis

All statistical analyses were completed in Program R v3.6.3 (R Core Team, Vienna, Austria) and the significance level for comparisons and testing was set at α = 0.05. We used a linear mixed effects model to test for differences in *mcrA* gene copies among treatments, with treatment as a fixed effect and tank ID as a random effect using the lmer function in the lme4 package (Bates et al., 2015). To test the hypothesis that *mcrA* gene copy differed among treatments at the end of the experiment, we computed pairwise comparisons using the emmeans function in the emmeans package (Lenth, 2021), adjusting pairwise *p*-values for multiple comparison using the Tukey method. Voltammetry data was processed using the data.table, and dplyr packages and visualized using ggplot2 (Wickham, 2016; Dowle and Srinivasan, 2020; Whickham et al., 2020).

## Results

### Sediments Maintain Anoxia Within 5 mm From the Sediment Water Interface

**FIGURE 2:**
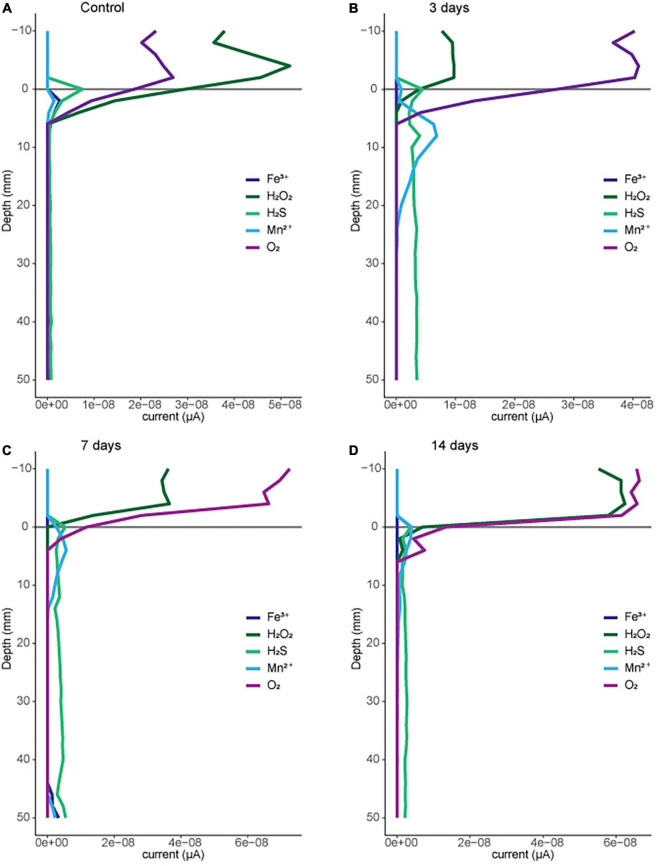
Geochemical profiles for representative disturbance treatments: **(A)** control, **(B)** 3 days, **(C)** 7 days, and **(D)** 14 days. Analytes measured using cyclic voltammetry on a Au-amalgam microelectrode as described in methods. Current above baseline given as a relative indicator of abundance for each species.

To evaluate the potential impacts of macroscopic bioturbation on the efflux of greenhouse gasses from sediments, we investigate the impacts of sediment disturbances (meant to mimic bioturbation by fishes at different frequencies in sediment mesocosms) on sediment redox conditions. We predicted that disturbances mimicking bioturbation could impact sediment redox conditions and consequently microbial activity. Redox geochemistry (including O_2_, Fe^2+^, Fe^3+^, Mn^2+^, and H_2_S) was measured electrochemically with depth in a replicate tank for each bioturbation treatment (Figure 2). Across each treatment the oxygen decreases from the aerated water column (<0 mm in Figure 2) to approximately 5 mm depth below the sediment surface. Variation in the depth profile likely results from the topography of the surface sediment which has been disturbed both by mechanical bioturbation and by benthic invertebrates (primarily oligochaetes) borrowing in the top few cm of sediment. The 3-day treatment had been bioturbated the day before oxygen profiles were measured, suggesting that any oxygen introduced to the deeper sediment *via* mechanical disturbance (20–30 mm) is consumed to below the detection limit (conservatively 10 μM) within a day of treatment. These profiles support that sediment at 1 cm (10 mm) and 3 cm (30 mm) are below detection at least 60% of the time over the treatment interval. The electrochemical profiles also provide evidence of other terminal electron accepting processes in the sediments, such as manganese reduction just below the sediment water interface, and where oxygen falls below detection. A consistent sulfide signal is observed with depth in each profile, with the strongest sulfide signal observed in the 3-day treatment. Overall, the profiles provide evidence for multiple anaerobic microbial processes in these sediment mesocosms within 0–5 mm below the sediment water interface and within 1 day of our disturbance treatments. To investigate the potential for microbial community structure to be affecting sediment processes we sequenced sediment for both disturbed locations (1 cm) and the depth just below the zone of disturbance (3 cm).

### Disturbance Frequency Did Not Significantly Impact the Structure of the Total Microbial Community Above and Below the Zone of Disturbance

**FIGURE 3:**
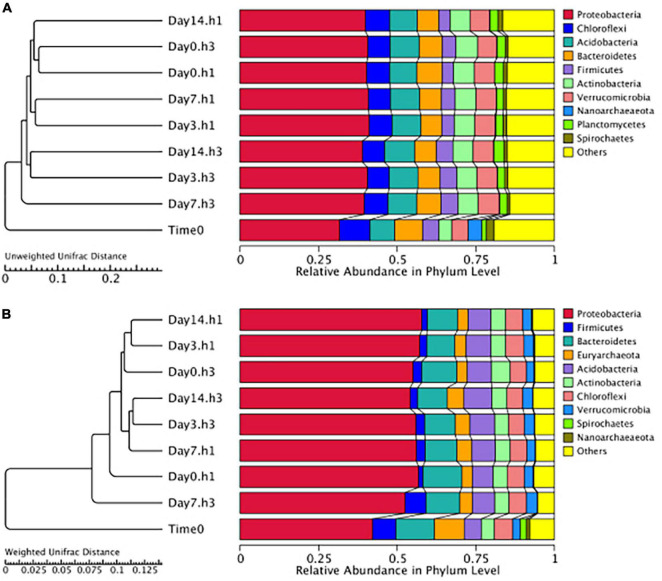
Hierarchical clustering of OTUs shows minimal differences in the total microbial community at the phylum level based on unweighted **(A)** and weighted **(B)** unifrac distances. Triplicate sediment samples for each treatment, including sediment height (h1 = 1 cm, h3 = 3 cm) and disturbance frequency (Day 3, 7, and 14) compared to no disturbance control (Day 0) and the original starting sediment composition (Time 0). Measurements support minimal significant differences in community structure across treatments and a lack of trend with bioturbation frequency.

Microbial composition did not significantly differ across sediments taken from the three different disturbance treatments (3-day, 7-day, and 14-day) compared to the control (0-day) samples (Figure 3). Branch lengths of less than 0.1 are observed in both weighted and unweighted Unifrac distances across the different treatment clusters, and no trend in clustering was observed based on treatment type including both sediment depth and disturbance frequency (Figure 3). These overall trends are supported by PcoA and PCA plots that demonstrate no significant clustering between treatment types or sediment heights but demonstrate a distinct clustering between the treatment and time zero data (Supplementary Figure 2). Additional analyses were performed to investigate the potential for enrichment of different OTUs based on either treatments or sediment depth, including MetaStat, LefSe, and DESeq (White et al., 2009; Segata et al., 2011; Love et al., 2014). No significant trends were observed in the OTUs that were enriched above 0.1%.

All treatment and sediment depths measured differed in abundance and diversity of certain taxa compared to the original homogenized mud used in construction of the sediment microcosms, our Time 0 control samples. Compared to the starting sediment microbial community (Time 0), all treatment communities shifted to a higher abundance (weighted) and diversity (unweighted) of Proteobacteria (Figure 3). While the number of OTUs present from the Chloroflexota, Firmicutes, and Euryarcheota did not change between the treatment and Time 0 samples, the relative abundance of these groups decreases in the disturbance treatments. Groups such as the Nanoarchaeota and Spirochetes decrease in both diversity and abundance (Figure 3).

**FIGURE 4:**
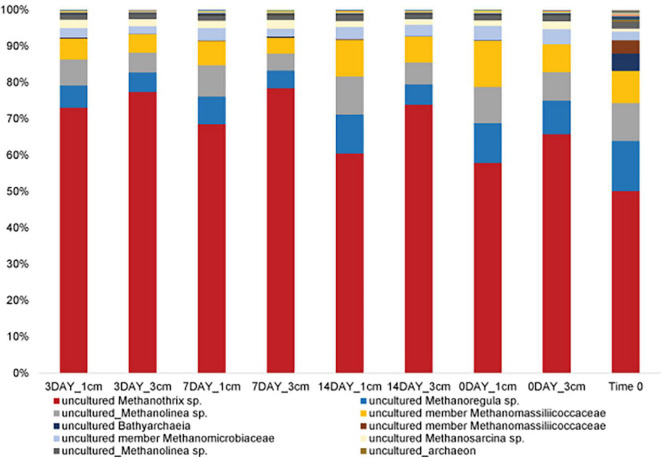
Methanogenic microbial community structure does not dramatically change with bioturbation frequency. Trend in percent of a putatively oxygen tolerant methanogenic phylotype [uncultured_Methanothrix sp. (OTU_4573)] as disturbance frequency increases, especially for 1 cm sediments. These methanogenic taxa, on average, comprise 3.3 ± 0.7% of the total microbial community.

**FIGURE 5:**
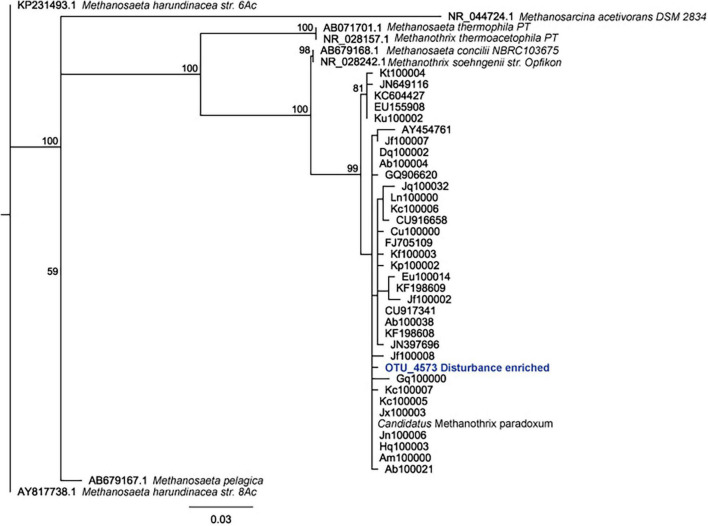
Phylogenetic tree demonstrating placement of *candidatis* Methanothrix paradoxum identified in this work (OTU_4573 Disturbance enriched).

**FIGURE 6:**
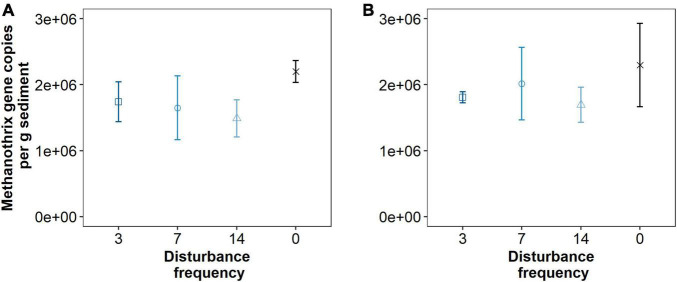
Quantitative PCR data for *ca.* Methanothrix paradoxum 16S rRNA gene copy numbers per gram sediment across different treatments at 1 cm **(A)** and 3 cm **(B)** depths. Triplicate reactions for samples taken from three replicate mesocosms analyzed for each data point (*n* = 9). Points are estimated marginal means and error bars are standard deviations.

Given that we hypothesized methanogenic Archaea would be affected by our disturbance treatments due to oxygen exposure, we investigated composition of the methanogenic microbial community specifically. As described, this includes members of the Euryarchaeota from the orders: *Methanosarcinales, Methanomicrobiales, Methanocellales* and *Methanomassiliicoccales*. Members of the Crenarchaeota lineages *Bathyarchaeia* and *Verstraetearchaeota* that have recently been shown to contain *mcrA* genes were also included in this analysis, though these were only minor community members in terms of OTUs recovered. The dominant methanogen phylotype observed across all treatments (55–70% of the methanogenic microbial community) was an uncultured *Methanothrix* phylotype (Figure 4). This OTU is the least abundant in the Time0 sample that also contains the highest overall diversity of distinct methanogenic OTUs (Figure 4). Interestingly, the *Methanothrix* phylotype identified is most closely related to a putatively oxygen tolerant and recently described *candidatis* Methanothrix paradoxum (Figure 5; Angle et al., 2017). The relative abundance of this organism in the methanogenic community increases with disturbance frequency especially within the disturbed region of sediment sampled (i.e., 1 cm; Figure 4). To determine if these changes in the *ca.* Methanothrix paradoxum were due to either faster growth or slower death of this phylotype, qPCR primers specific to this OTU were designed. No significant changes in 16S rRNA copy numbers for *ca.* Methanothrix paradoxum across any of the sediment depths or treatments was noted (Figure 6). These values were all significantly lower than the values observed in the original Time 0 sediment treatment (3.6 × 10^6^ ± 3.9 × 10^5^ copies per gram sediment). These data suggest that this *Methanothrix* phylotype is more resilient (i.e., doesn’t die as quickly) in shallower sediments and with increased oxygen exposure compared with the other taxa present.

### Disturbance Negatively Impacts Methanogen Abundance and May Select for Oxygen Tolerant Methanogenic Microbes

**FIGURE 7:**
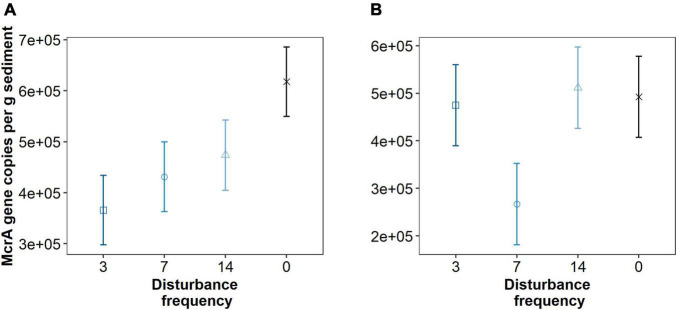
Quantitative PCR data for *mcrA* gene copy numbers per gram sediment across different treatments at 1 cm **(A)** and 3 cm **(B)** depths. Triplicate reactions for samples taken from three replicate mesocosms analyzed for each data point (*n* = 9). Points are estimated marginal means and error bars are SE. Means that do not share letters significantly differ (α = 0.05). A significant linear trend between increasing disturbance and decreasing methanogen abundance at 1 cm depth (df = 7.9, *t* = 2.63, *p* = 0.031), but not at 4 cm depth (df = 8.0, *t* = 0.782, *p* = 0.456), was observed.

Though we see minimal shifts in the total microbial community, we hypothesized that disturbance frequency could result in a reduced abundance of methanogenic organisms. Using the *mcrA* gene copies as a proxy for methanogen abundance (Figure 7), we observed a significant linear trend between increasing disturbance and decreasing methanogen abundance at 1 cm depth (df = 7.9, *t* = 2.63, *p* = 0.031), but not at 3 cm depth (df = 8.0, *t* = 0.782, *p* = 0.456). No trend was observed analyzing a cubic or quadratic fit to the data. Though the trend in decreasing *mcrA* copy numbers with increased disturbance was significant for the disturbed region of sediment, pairwise comparison of individual treatments was not different at either sediment depth. These trends were consistent whether the data was analyzed per gram sediment, per volume total DNA, or per ng DNA recovered from the sediment.

We also investigated the potential impact of our disturbance treatments on methanotrophic microbes at 1 cm and 3 cm sediment depths. Consistent amplification of the particulate methane monooxygenase gene *pmoA* was only observed in 1 cm sediment samples. No amplification of the soluble methane monooxygenase *mmoX* was detected in any sample. Quantification of the *pmoA* gene at 1 cm generated very low gene abundances (two orders of magnitude lower than *mcrA* gene copies) that were highly variable (Supplementary Figure 3). As such, no statistically significant differences were noted across the disturbance treatments in methanotroph abundance.

## Discussion

The impacts of bioturbation on sediment geochemistry and microbial processes are dependent on the magnitude and frequency of the sediment disturbance, though the explicit relationships between different modes of bioturbation and specific processes remain poorly characterized. As shown in our previous work, we investigated the relationship between sediment disturbances mimicking benthic feeding fish and greenhouse gas production. We found that methane emissions were enhanced at intermediate disturbance frequencies, and this was driven by the ebullition of methane (Figure 1; Booth et al., 2021). Given that the physical disruption of the sediment was shown to drive the majority of methane ebullition from sediment [up to 75% of the total emissions (Booth et al., 2021)], we predicted that the intermediate disturbance frequencies balanced production of methane (by strictly anaerobic methanogens), with ebullition that is enhanced by mechanical disturbance, while limiting residence time of methane and therefore consumption. This work sought to test the hypothesis that disturbance impacts the methane producing community, most likely through the periodic introduction of oxygen into sediments that were previously anoxic.

Voltametric measurements of sediment demonstrate that oxygen is consumed within the first 5 mm of sediment for all bioturbation frequencies. However, one can reasonably assume that mechanical bioturbation would have oxygenated the subsurface through the depth of mixing (at least 20 mm). Our measurements demonstrate that the oxygen profile is re-established within 1-day post-disturbance with oxygen falling below detection at 5 mm. Future work is planned to more rigorously characterize the timing of redox changes post-bioturbation at shorter timescales. Though the specific magnitude and timing of oxygen introduction and subsequent consumption back to a steady-state gradient have yet to be characterized, even short oxygen exposures have been shown to inhibit a wide range of methanogenic species (Zinder, 1993; Thauer et al., 2008). Most of the biochemical pathway involved in methanogenesis is sensitive to oxygen, as it contains highly reactive metal containing co-factors which are either easily oxidized or can result in Fenton reactions which produce destructive free radicals (Cedervall et al., 2010). While some species have been shown to contain strategies for dealing with the presence of oxygen, often its presence results in a complete shutdown of metabolic activity (Kato et al., 1993; Yuan et al., 2009; Freitag et al., 2010). In culture, multiple taxa have been shown to shut down methanogenesis for up to 3 days (Kiener and Leisinger, 1983) upon oxygen exposure. In the environment, a shutdown in metabolic activity would impact an organisms’ ability to grow and/or compete with other organisms for nutrients. This is consistent with the trend we observed in this work, with methanogen abundance decreasing with an increase in disturbance frequency as this would also increase the frequency of oxygen exposure to methanogenic organisms in the impacted area (i.e., 1 cm depth) compared to a region not impacted by disturbance (i.e., 3 cm).

The total microbial community was not significantly altered by the different disturbance treatments, however there was a trend observed in the methanogen-specific community that demonstrated a marked relative increase in a putatively oxygen-tolerant methanogen phylotype, *candidatus* Methanothrix paradoxum (Angle et al., 2017). Given that 16S rRNA abundance of *ca.* Methanothrix paradoxum did not change on an absolute scale (Figure 6), this suggest that other methanogen populations were more strongly impacted by the disturbance treatments. This phylotype was previously identified from sediments in an oxic and methane producing wetland, in Old Woman Creek. Other members of the *Methanothix* genera (previously *Methanosaeta*) are known for acetoclastic methanogenesis, often with a high affinity for acetate (Patel and Sprott, 1990; Ma et al., 2006). *Candidatus* M. paradoxum was named for the conundrum of it’s ability to make methane under functionally oxic conditions (Angle et al., 2017). Metatranscriptomic work for Old Woman Creek supported the active transcription of methanogenesis genes from oxic sediments and showed no evidence of oxygen exposure/stress. Nonetheless, given the genomic features, the authors suggest that it is unlikely that *candidatus* M. paradoxum is immune to oxygen, though perhaps can generate anoxic niches in these functionally oxic systems that facilitate generation of methane. Alternatively, these organisms could contain other not well characterized strategies for dealing with oxygen exposure that allow them to persist and actively generate methane in the top few cm of sediment impacted by disturbance.

One caveat to this work is that fish disturbance of sediments is linked to feeding behaviors and thus linked to the removal of invertebrates As reported in our prior work (Booth et al., 2021), the invertebrate communities were composed of oligochaetes, with relatively few stonefly larvae (Plecoptera) and snails. The majority of the invertebrate communities were composed of oligochaetes and no difference in the invertebrate communities were demonstrated due to disturbance (Booth et al., 2021). However in recent work looking at the impact of different fish and macroinvertebrate impacts on greenhouse gas emissions, invertebrate feeding fishes that reduced the number of tubifex worms were shown to increase *mcrA* gene copies, likely by reducing oxygen exposure from worm activities in the top cm of sediment (Colina et al., 2021). Notably fish activities in the Colina et al. (2021) work, did not impact ebullition of methane. This likely speaks to the minimal sediment disturbance due to the fish species and sizes used, which further speaks to the potential importance of degree of disturbance linked to fish activities. Additionally, the specific methanogen species were not quantified in the Colina et al. (2021) work and thus insights into their susceptibility to oxygen which may also play a role in the *mcrA* copy number dynamics (indicative of methanogen populations) are difficult to determine. For example, we highlight that the top few centimeters of sediment is an active and important region for methanogenic activity and makes significant contributions to the overall methane emissions in sediment. In this work no effects on the community or methanogenic population at 3 cm depths were noted, indicating that the quantifiable differences in methane emissions between disturbance treatments was due to microbial activity in the shallow sediment. As a difference was noted at 1 cm depth, this supports the important contributions of shallow methanogen populations to methane production, however the composition of the methanogenic microbial community and their corresponding tolerance to oxygen will likely impact the methanogenic activity in these shallow sediments.

Overall, this work supports the hypothesis that mechanical disturbance, or more particularly, oxygen exposure caused by bioturbation, can affect the methanogenic community of freshwater sediments and impact methane emissions. However, this work also supports that multiple factors are impacting methane emissions. We found a linear trend between the decrease in methanogens and an increased frequency of disturbance. This matched the trend seen in greenhouse gas emissions for high to moderate frequency disturbances. Specifically, 3 days of disturbance had significantly lower methane emissions than 7 days. However, the later time points had a trend in higher methanogen populations, but lower emissions than the 7-day treatment. We predict that the longer residence time of methane supported more methane diffusion (relative to ebullition) and stimulated the activity of methanotrophic populations which acted to reduce total methane emissions. We were unable to quantify a difference in the methanotrophic population across treatments at 1 cm sediment depths. However, this does not rule out that methanotrophic organisms in surface sediments with more consistent oxygen exposure are driving the enhanced methane consumption at lower disturbance frequencies. This hypothesis will be more fully tested in our follow up work which will broaden the range/depths of sediment samples tested, expand the frequencies of disturbance, and look more directly at the impacts of fish-specific bioturbation. Though this work is preliminary, given the interesting patterns observed and the likelihood of multiple processes impacting the methane emissions, it points to developing a mechanistic framework to better understand the impacts of processes like disturbance, bioturbation and sediment microbial ecology on the heterogenous nature of methane emissions in a range of freshwater ecosystems.

## Data Availability Statement

The datasets presented in this study can be found in online repositories. The nucleic acid data presented in this study are deposited in the NCBI repository, under bioproject number PRJNA766591 with associated SRA and biosample information.

## Author Contributions

AR helped plan the experiments, supervised lab work, mentored MU and LT, performed the data analysis and interpretation, and authored the manuscript. MU performed the mesocosm studies, collected the samples, extracted nucleic acids, helped with the data analysis, and provided the editorial comments. LT supervised nucleic acid work, helped with the data analysis, and provided the editorial comments. JS performed the redox sediment measurements, performed the data analysis and interpretation, and provided the editorial comments. GD mentored JS, helped with the data analysis and interpretation, and provided the editorial comments. MB designed the experimental set up, acquired funding, mentored MU, helped with the data analysis and interpretation, and provided the editorial comments. All authors contributed to the article and approved the submitted version.

## Conflict of Interest

The authors declare that the research was conducted in the absence of any commercial or financial relationships that could be construed as a potential conflict of interest.

## Publisher’s Note

All claims expressed in this article are solely those of the authors and do not necessarily represent those of their affiliated organizations, or those of the publisher, the editors and the reviewers. Any product that may be evaluated in this article, or claim that may be made by its manufacturer, is not guaranteed or endorsed by the publisher.

## Footnotes

1
https://github.com/Apperception-Group/FREEDOX

2
www.idtdna.com/scitools/Applications/RealTimePCR

3
www.idtdna.com/scitools/Applications/RealTimePCR
