# Supplementary material for: Sediment Disturbance Negatively Impacts Methanogen Abundance but Has Variable Effects on Total Methane Emissions
Source: Front Microbiol. 2022 Feb 21;13:796018. doi: 10.3389/fmicb.2022.796018 (PMC8899539; doi:10.3389/fmicb.2022.796018)
Supplement: Supplementary file 1 [file Table_1.DOCX]

Supplementary Material

Sediment disturbance negatively impacts methanogen abundance but has variable effects on total methane emissions

**Annette Rowe^1*^, Megan Urbanic^1^, Leah Trutschel^1^, John Shukle^2^, Greg Druschel^2^, Michael Booth^1^**

^1^Department of Biological Sciences, University of Cincinnati, Cincinnati, Ohio, USA

^2^Department of Earth Sciences, Indiana University Purdue University Indianapolis, Indianapolis, Indiana, USA

*** Correspondence:**Corresponding Author
annette.rowe@uc.edu

Keywords: methanogenesis, bioturbation, freshwater sediment, ebullition, greenhouse gases

# Supplementary Materials and Methods

## Methane Quantification

Methane ebullition was measured using inverted gas funnel traps constructed from a 165 mm diameter plastic funnel glued to a 60-mL gas-tight syringe (illustrated in supplemental Figure S1 and described in Booth et al., 2021). The syringe and funnel were glued using a waterproof adhesive and the syringe was closed using a 3-way luer-lock stopcock. The funnel was completely submerged (30-40 mm above sediment water interface) and the syringe was filled with water—to be displaced by any ebullition in the region covered by the funnel. Each tank consisted of three traps equating to 0.064 m^2^ surface area. During scheduled disturbances, the bubble traps were elevated to 40 mm to allow for any bubbles released during disturbance to be captured effectively. Gas volumes were quantified using the graduation marks on the syringes, and recorded pre and post disturbance treatments. Gas composition was quantified in up to three replicate composite gas samples that were placed in pre-evacuated 5 mL glass vials (Exetainers, LabCo, Wales, U.K.) equipped with a PTFE silicon septa stacked on a chlorobutyl rubber septa. CH_4_ and CO_2_ were measured on a Bruker 450 or Shimadzu GC-2014 gas chromatograph equipped with a methanizer and flame ionization detector. Ebullition samples were analyzed using a minimum of one 5‐point standard curve bracketing the expected concentrations for each analyte. Standard curves were created using certified or primary standards, had a minimum r^2^ of 0.990, and standard checks were analyzed throughout analytical runs. Minimum detection limits were 25 and 0.25 ppm for CO_2_ and CH_4_, respectively.

Diffusive gas measurements were taken at two-to-three-week intervals using an Ultra-Portable Greenhouse Gas analyzer (Los Gatos Research, San Jose, CA, USA) as described previously (Booth et al., 2021). A sealed plexiglass chamber was set on top of the tank rim and connected to the gas analyzer via 0.32 cm i.d. tubing. Over a 5-10 min deployment the gas analyzer continuously recirculated the chamber headspace, recording H_2_O, CH_4_and CO_2_ partial pressures at 1 s intervals. Diffusive emission rates were calculated as CH_4_‐D = (Δc/Δt)(V/A)(P/RT) where Δc/Δt is the rate of change (ppmv h^‐1^) of CH_4_ in the chamber headspace, V is the chamber volume (L), A is the area of the water surface enclosed by the chamber (m^2^), P is the pressure (atm) inside the chamber (assumed to be equal to atmospheric pressure), R is the universal gas constant, and T is the air temperature (K). CH_4_ rate of change in the chamber headspace was quantified using a linear model. Measurements occasionally coincided with ebullition events, however, if this occurred, measurements were removed from analysis and/or re-measured.

# Supplementary Data

Supplementary Data: OTU table (attached)

# Supplementary Figures and Tables

## Supplementary Figures


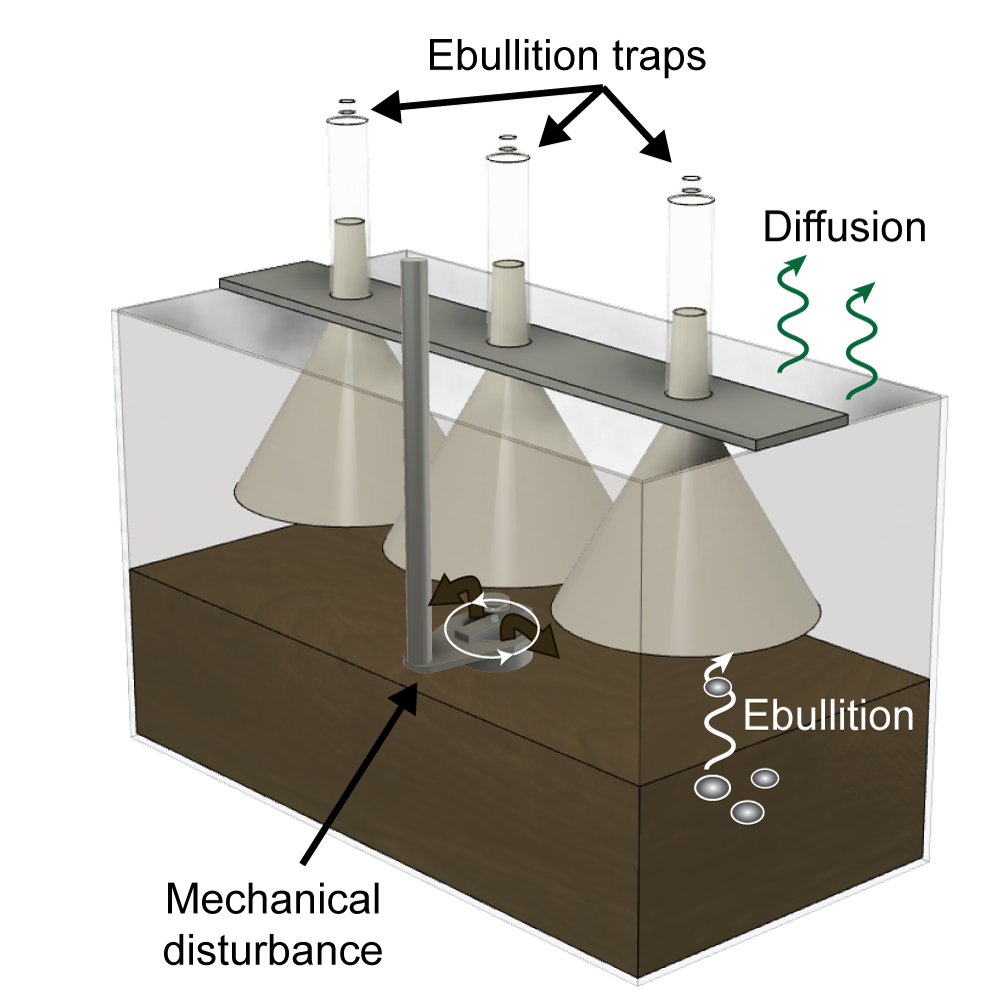


**Figure S1.** Schematic of aquarium set up for study of frequency of mechanical disturbance in relation to greenhouse gas emissions via ebullition or diffusion. A “bioturbator” was constructed to disrupt sediment analogous to the disruption caused by benthic feeding fish.


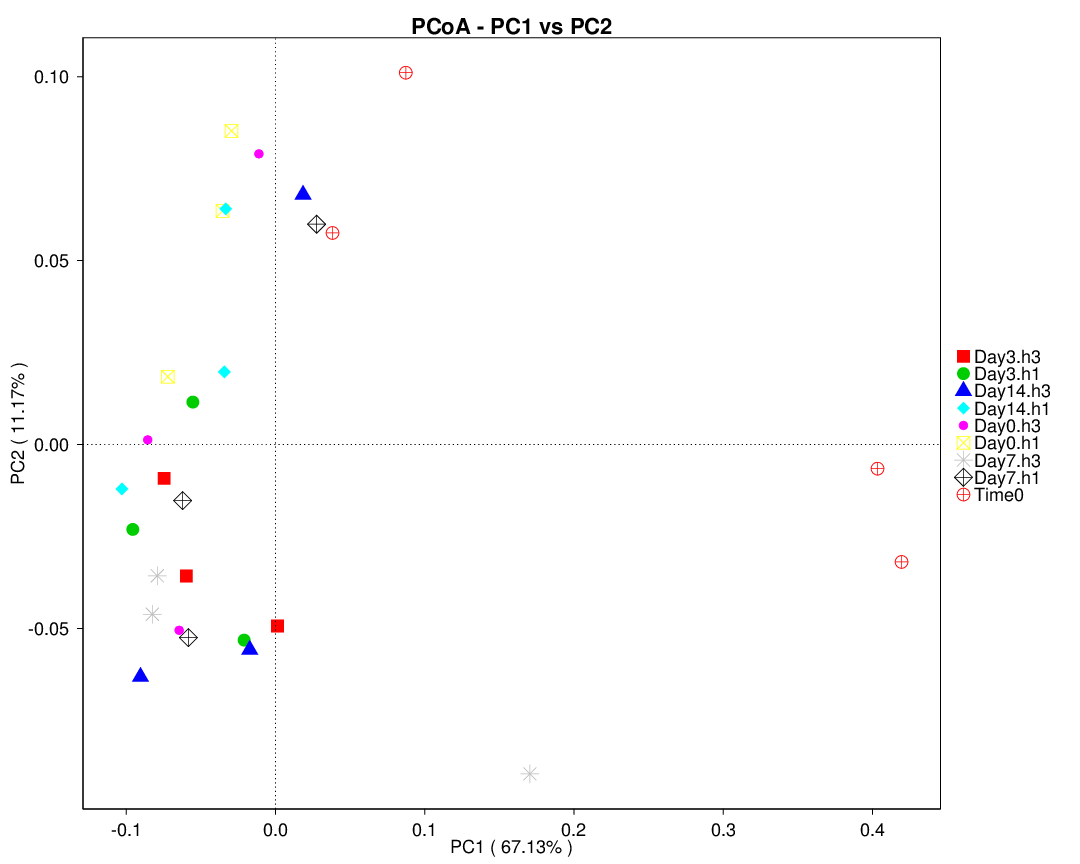

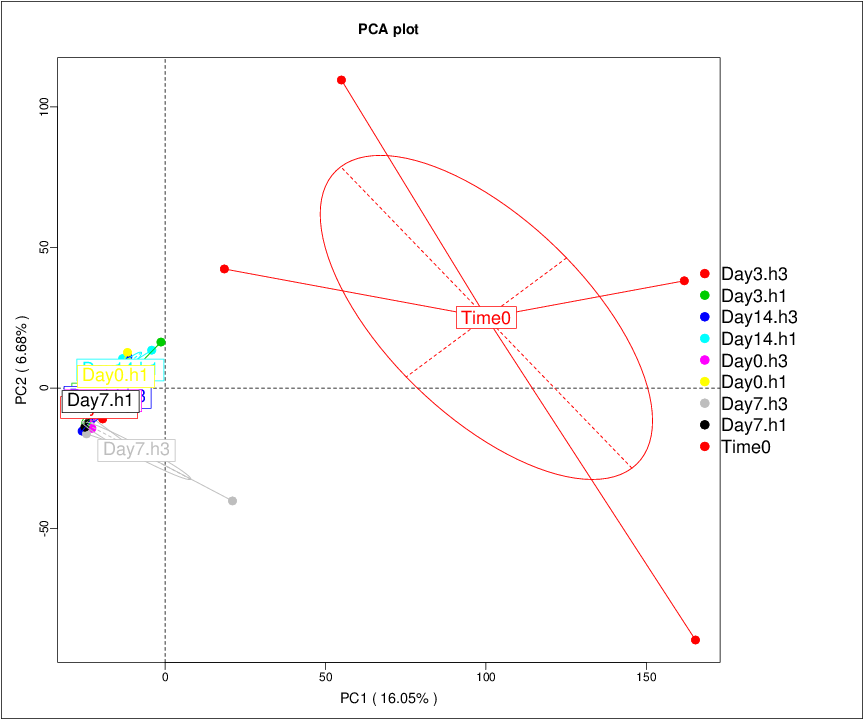


**Figure S2**. Principle coordinate analysis (PCoA) and principal component analysis (PCA) for 16S rRNA amplicon sequencing generated for sediment samples at different depths (h1 [1 cm] and h3 [3 cm]) and different frequencies of disturbance (0, 3, 7 and 14 days) compared to the starting homogenized sediment community composition (Time 0). No significant cluster observed between treatments with the exception of Time 0 and all disturbance treatment clusters.

**Figure S3.** Quantitative analysis of *pmoA* gene copies per gram sediment at 1 cm depth (Day 0,3,7 &14) for disturbance treatments and for the control, pre incubation sediment sample (Time 0). Error bars represent standard deviations of three biological replicates (n = 3) and demonstrate a lack of statistically significant differences be tween treatment and with control sample.

**
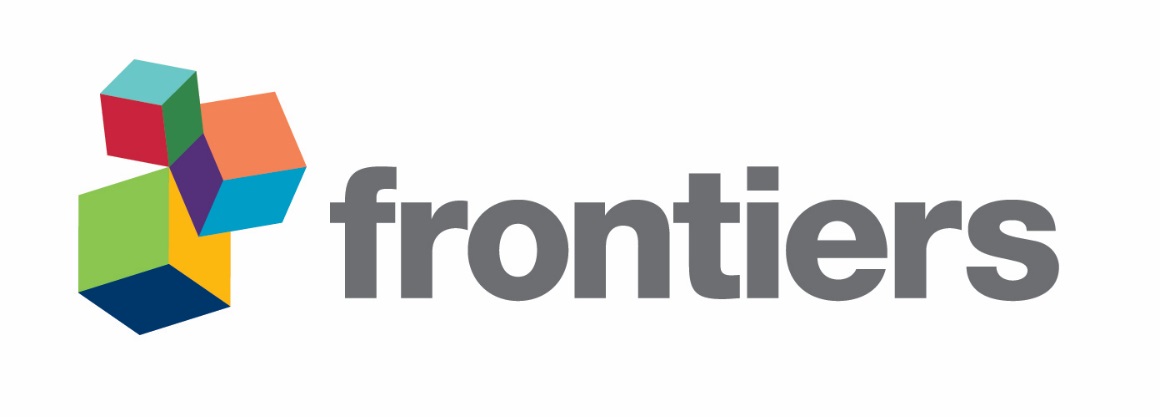
**
